# Is the Gut Microbiota a Neglected Aspect of Gut and Brain Disorders?

**DOI:** 10.7759/cureus.19740

**Published:** 2021-11-19

**Authors:** Yuvaraj Balan, Archana Gaur, Varatharajan Sakthivadivel, Bhushan Kamble, Raja Sundaramurthy

**Affiliations:** 1 Department of Biochemistry, All India Institute of Medical Sciences, Bibinagar, Bibinagar, IND; 2 Department of Physiology, All India Institute of Medical Sciences, Bibinagar, Bibinagar, IND; 3 Department of Internal Medicine, All India Institute of Medical Sciences, Bibinagar, Bibinagar, IND; 4 Department of Community and Family Medicine, All India Institute of Medical Sciences, Bibinagar, Bibinagar, IND; 5 Department of Microbiology, All India Institute of Medical Sciences, Bibinagar, Bibinagar, IND

**Keywords:** gut-brain axis, neurodegenerative diseases, inflammatory bowel disease, gut dysbiosis and diseases, gut microbiota

## Abstract

The gut microbiota is a quickly developing bacterial ecosystem with biodiversity. It is an adaptive immunity that varies with food intake, environmental conditions, and human habits, among other factors. Various external stimuli, such as drugs, can influence the gut microbial environment and lead to gut dysbiosis. Recently, gut dysbiosis has been identified as an important factor that leads to several diseases either by the released metabolites or by the gut neuronal connection. In brain disorders, gut dysbiosis is involved in neuropsychiatric manifestations, including autism spectrum disorder, anxiety, and depression by interfering with neurotransmitter homeostasis, and neurodegenerative diseases, such as Alzheimer’s disease and Parkinson’s disease by releasing abnormal metabolites from the gut. Gut dysbiosis has been documented in gut disorders, including inflammatory bowel disease or irritable bowel syndrome. Immune cells in the gut are modulated by external factors such as stress, diet, and drugs to produce inflammatory cytokines, including interleukins (IL-4, IL-6, IL-17, IL-23, etc.). Inflammatory cytokines lead to a cascade of events, which lead to various ailments in the bowel. Beneficial bacteria in the form of probiotics ameliorate the condition and have healthful effects in disease conditions. This warrants further research to identify newer therapeutic strategies for diseases that cannot be cured or are difficult to treat.

## Introduction and background

The human body is inhabited by bacteria, archaea, viruses, and eukaryotic microbes. The organization of microorganisms harboring the gastrointestinal (GI) tract (GIT) is collectively called the “gut microbiome.” The gut microbiome of an individual is specific to that individual, often known as “the microbial signature” [1]. The gut microbiota is diverse, even in healthy individuals [2]. The symbiotic association between microbes and the human body keeps the body healthy and protects against diseases [3,4]. The development of this gut microbiome has been thought to originate only after birth, but recent evidence suggests that the development starts in the fetal stage itself [5,6]. The GIT appears to be involved in many diseases, such as depression, Alzheimer’s disease, and Parkinson’s disease, and a change in the gut microbiome can be noted in various diseases [7-9]. The gut-brain axis (GBA) and its role in feeding and satiety are known facts. Evidence suggests that the neurotransmitters involved in the GBA can be manipulated by the gut microbiome [10,11]. The neurotransmitters, along with the gut microbiome, assist in monitoring and amalgamating gut functions along with emotional and cognitive centers of the brain. They also play a major role in immune system activation, intestinal permeability and motility, and enteroendocrine signaling [10]. The complex dialog between the GBA is mediated by neural, metabolic, endocrine, and immune responses to diverse environmental cues, including diet and components of the intestinal microbiota [12]. Hence, the gut microbiome plays a role in metabolic functions, protection against pathogens, and immune functions; thus, it contributes to normal physiology [2,13,14]. The derangement in the gut microbiome and its harmonical relations with normal physiology is collectively termed “gut dysbiosis.” The concept of gut dysbiosis [15] and its role in diseases/ailments of the brain and intestine are reviewed in this article. This review also aims to explore future research perspectives of the gut microbiome in the management of these diseases.

## Review

Gut microbiota

**Figure 1:**
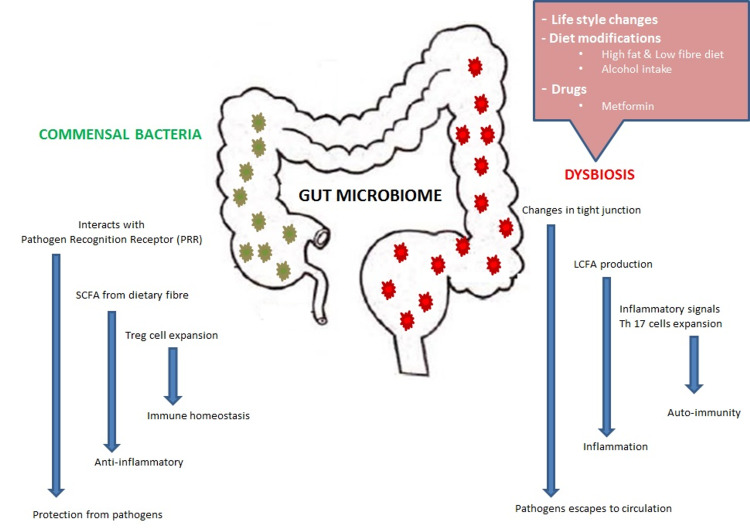
Gut microbial ecology and disturbances. LCFA: long-chain fatty acid; SCFA: short-chain fatty acid; Th17: helper T cell 17; Treg: regulatory T cell

Gut microbial ecology is diverse and complex. The gut microbial system comprises dynamic microbes ranging from beneficial microbiota to opportunistic pathogens. Commensal bacteria colonize the intestine immediately after birth. As humans mature, more bacteria colonize the intestine. On average, a healthy adult GIT harbors approximately 1,000 bacterial species. Firmicutes, Bacteroidaceae, Lachnospiraceae, Actinobacteria, Prevotellaceae, and Ruminococcaceae are the dominant group of bacterial species [7]. Gut microbial species are important for the maintenance of normal human health. For example, they help in the digestion of macromolecules, synthesis of vitamins, metabolism of bile acids, etc. However, the composition of bacterial species varies with lifestyle changes, diet modifications, and medication use [12]. Dysbiosis occurs when there is a functional or compositional imbalance in the gut microbiome. Figure 1 depicts gut microbial ecology and disturbances.

Physical Inactivity

In obesity, the number of Firmicutes increases compared to abundant Bacteroides. In general, Firmicutes extract more energy from dietary components and lead to more adiposity. This finding was confirmed by Ley et al. who analyzed stool samples from twelve obese and five lean individuals. They also performed genetic sequencing to determine different bacterial strains. Obese individuals had more Firmicutes and fewer Bacteroidetes than lean individuals. Further, when obese volunteers followed a weight-loss diet for months, there was a significant increase in Bacteroides, but not equal to lean individuals [16]. In another study, there was a 20% reduction in Bacteroides in individuals with high-calorie intake, which was directly related to weight gain [17].

Diet Modifications

Recent data from a human study revealed that dietary factors directly influence the composition of the gut microbiota. Particular importance is given to the westernization of diet, which is high in animal fat and proteins and lacks dietary fibers. Saturated fat and animal-derived proteins increase cholesterol levels, increase Firmicutes and Enterobacteriaceae, and reduce Bacteroidetes, such as in an obese gut microbiome environment, which increases the propensity for inflammation, thrombosis, etc. A vegetable-based diet is accompanied by an increase in Prevotella and Firmicutes, which are involved in the degradation of dietary fibers, as well as by an increase in anti-inflammatory and antianxiety activities [18]. Fructose feeding results in a significant reduction in Bacteroidetes and an increase in pathogenic Helicobacteraceae colonies. Alcohol consumption results in increased Bacteroidetes and Firmicutes and decreased Faecalibacterium [19]. This finding correlated well with several other studies. Moreover, alcohol intake increases intestinal permeability through which intestinal metabolites and bacteria enter the systemic circulation, causing organ damage and inflammation.

Dietary intake of herbal products, such as Rhizoma Coptidis, modulates the gut microbiota and reduces cholesterol levels and inflammation. A high-fiber diet increases Prevotella, Bifidobacterium, and Eubacterium spp., which degrade dietary fiber to produce short-chain fatty acids (SCFAs) which are anti-inflammatory. This was confirmed by another study, which showed that SCFA-propionic acid protects the mouse model from autoimmune colitis and increases neuronal plasticity. In the gut, fermented foods, yogurt, and natural marine products increase health-friendly bacteria, such as *Lactobacillus* and *Bifidobacterium* [20,21].

Drugs

With the increased usage of drugs, the gut microbiome is impacted by the direct effects of drugs. Different drugs have variable implications on microbial flora and thus play a role in dysbiosis. Nonsteroidal anti‐inflammatory drugs (NSAIDs), antipsychotics, and antidiabetic drugs change the microbial flora [22]. For example, Wang et al. demonstrated increased Proteobacteria and Bacteroides and decreased Firmicutes and *Lactobacillus* after usage of NSAIDs [23]. This finding was confirmed in a human study by Rogers et al. who found that *Prevotella* spp. and *Bacteroides* spp. were abundant in the stools of 155 adults who had taken NSAIDs compared to individuals not taking NSAIDs [24]. Recent animal and human studies have shown that the administration of metformin alters the intestinal microbiome environment. Metformin intake increased *Escherichia* spp. and *Lactobacillus* and decreased *Intestinibacter* [25].

Gut dysbiosis in diseases of the central nervous system

Although gut microbiome ecology and changes have been studied in the past decades, specific changes in the microbiome have been recently implicated in certain diseases. This review attempts to explore the possible mechanism by which changes in the gut microbiota lead to diseases of the central nervous system (CNS).

Gut-Brain Axis

**Figure 2:**
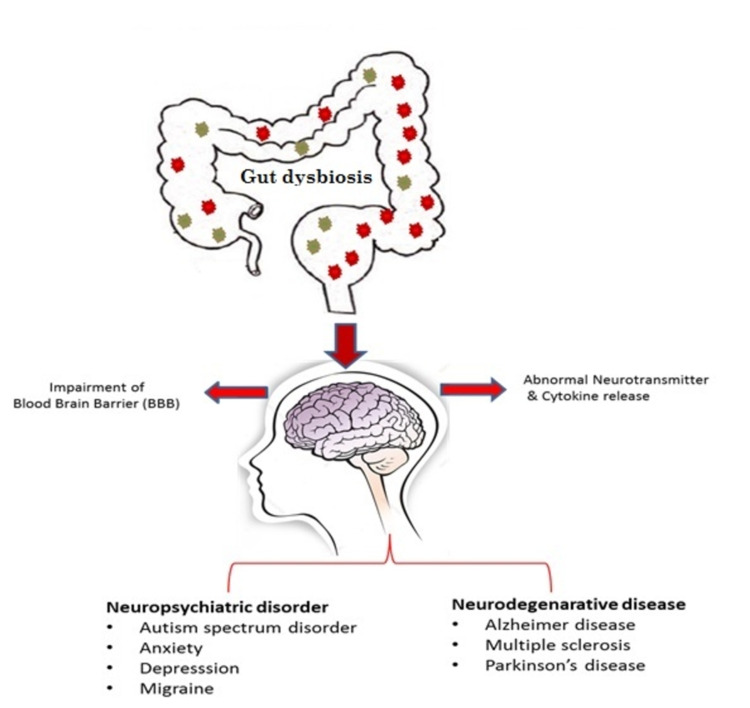
Gut dysbiosis in disorders of the brain.

There are no definitive treatments for most neurological diseases. Numerous studies have been performed to understand brain physiology and molecular mechanisms in diseases. Several factors have been found to play a role, ranging from environmental factors to genetic makeup. Most of these factors interface with the changes in the gut microbiome. Gut dysbiosis releases metabolites and cytokines that trigger inflammation, affect the blood-brain barrier (BBB) and brain volume, and can act as false neurotransmitters, resulting in altered brain physiology and neuronal function in neurological disorders, which may be a demyelinating or neuropsychiatric illness. Figure 2 depicts gut dysbiosis in disorders of the brain.

Neurodegenerative diseases

Parkinson’s Disease

**Figure 3:**
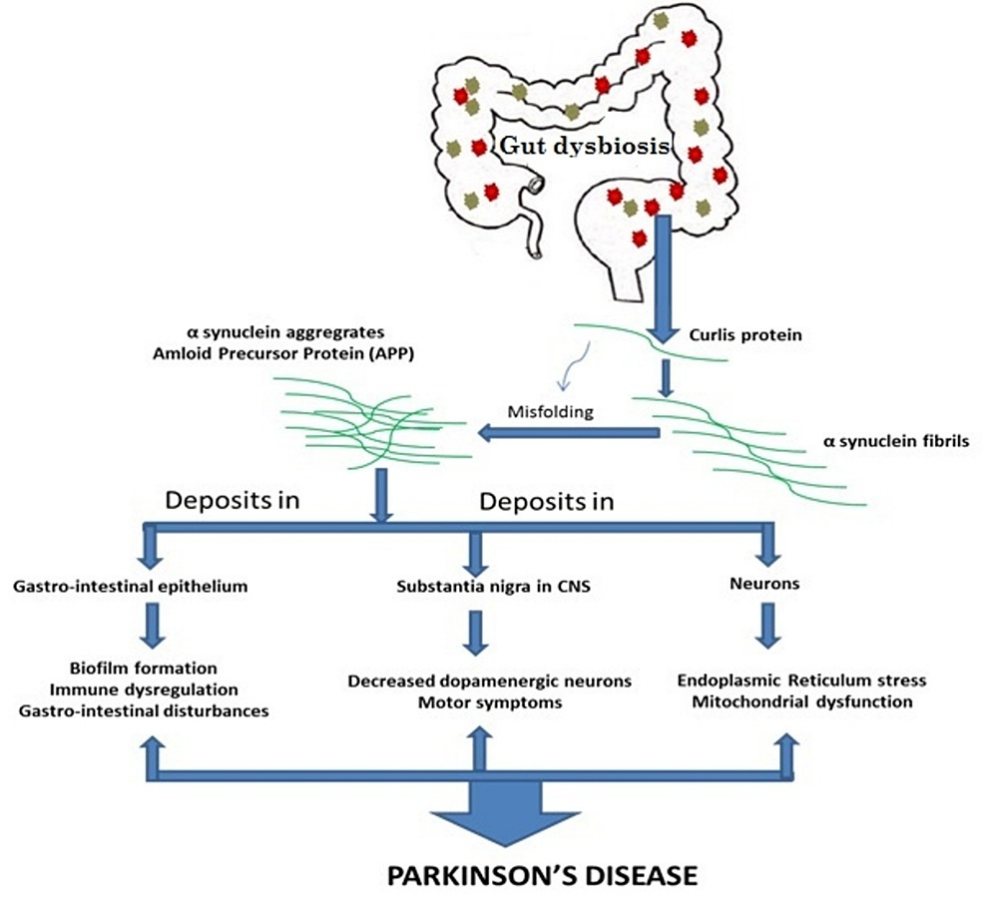
Role of gut dysbiosis in Parkinson’s disease CNS: central nervous system

Parkinson’s disease is characterized by muscle weakness and gait abnormalities. Scientists initially thought that it was related to the brain. Patients with Parkinson’s disease often complain of GI symptoms, such as constipation and inflammatory bowel disease (IBD)-like symptoms, which led scientists to explore the GI system in Parkinson’s disease [26,27]. Baark et al. found synuclein deposits in the brain and GI nervous tissue. This confirmed the suspicion, and scientists started to inspect the GI system. They found a bacterial strain in the gut (*Escherichia coli*) that produces a protein termed curli. Curli proteins coalesce to form synuclein proteins. They also cause synuclein and other proteins to misfold. Synuclein produced from cells acts as a monomer [28]. Multiple monomers join to form a fibril. Fibrils with misfolded protein aggregate to form amyloid fibrils that deposit in the neurons to cause neurodegeneration [29]. Synuclein also deposits in dopaminergic neurons in the brain, resulting in motor symptoms. Curli protein also forms a biofilm in the gastroepithelial tissue, which can prevent human immune defense, resulting in constipation and symptoms of IBD [30]. Figure 3 depicts the effect of gut dysbiosis in Parkinson’s disease.

Alzheimer’s Disease

Alzheimer’s disease is characterized by progressive dementia and cognitive and motor function impairment. The underlying etiology of the disease is debatable. Several theories, such as genetics, neuroinflammation, vascular degeneration, and calcium and energy balance disorder, have been proposed. However, therapies targeting these theories have not been fruitful. Therefore, there is an urgent need for new therapies to prevent and treat this devastating neurological condition [31].

A change in the gut microbiome is one of the crucial underlying factors for developing Alzheimer’s disease [8]. Brandshield et al. reported increased Firmicutes and decreased Bacteroidetes in a mouse model of Alzheimer’s disease [32]. This finding was confirmed by Vogt et al. who showed increased Firmicutes and Bacteroidetes and decreased *Bifidobacterium *[31]. In another human study of Alzheimer’s disease, Cattaneo et al. showed an increase in the number of *Escherichia*/*Shigella* and a decrease in *Eubacterium rectale* and *Bacteroides fragilis* [33]. In summary, in Alzheimer’s disease, there is a definite alteration in the gut microbiome, leading to gut dysbiosis. A decrease in anti-inflammatory bacterial species, such as *Bifidobacterium*, and an increase in proinflammatory species, such as Bacteroidetes and Firmicutes, were seen. This inflammatory microbiome releases cytokines and inflammatory markers into circulation. This disrupts the BBB and leads to an inflammatory state in the CNS, as seen in Alzheimer’s disease.

The hallmark pathogenic feature of Alzheimer’s disease is the accumulation of amyloid-β (Aβ) and tau protein aggregates. The gut microbiome produces various metabolites on food fermentation. One metabolite is SCFA, which can cross the BBB and interfere with Aβ and tau proteins, causing aggregates [34]. This was confirmed in both in-vivo and in-vitro studies. In a large meta-analysis by Xu and Wang, SCFA increased inflammation in the brain and caused aggregation of Aβ and tau proteins [35]. In an in-vivo study by Ho et al., the gut microbiome produced metabolites, such as isovaleric acid, propionic acid, and butyric acid, which caused Aβ polymers in a dose-dependent manner and Aβ aggregates. However, valeric acid administration inhibited Aβ polymer formation [36].

In addition, the gut microbiome could produce neurotransmitters, such as dopamine, acetylcholine, noradrenaline (*Escherichia* spp. and *Bacillus* spp.), serotonin, histamine (*Enterococcus* spp.), and gamma-aminobutyric acid (GABA; *Bifidobacterium* spp.). These neurotransmitters affect the host’s well-being and maintain homeostasis [11]. Gut dysbiosis causes altered neurotransmitter release, and some can act as false neurotransmitters in the brain, leading to behavioral changes, mood swings, sleep deprivation, depression, and increased anxiety, as seen in Alzheimer’s disease [37].

Neuropsychiatric diseases

Autism Spectrum Disorder

Autism spectrum disorder (ASD) is a developmental disorder characterized by anxiety, depression, forgetfulness, decreased memory, abnormal social behavior, and stress. Complex mixtures of events urge patients to distance themselves from society and decrease their communication behavior. Patients’ cognitive behavior, mood, and memory changes were initially thought to be due to a developmental disorder that affects the brain. However, recent studies have suggested that the gut microbiota can influence mood and behavioral changes starting from infancy to adulthood [38]. The microbiome starts colonizing the gut immediately after birth and starts connecting to the brain in the developmental process. Any inflammation or hindrance during the developmental process results in defective cognition, mood, memory, and abnormal behaviors [39].

Studies on mice have suggested that an infection during pregnancy results in a cascade of events. Segmented filamentous bacteria from the mother’s gut can induce an inflammatory event that further stimulates inflammatory helper T cells (Th17) cells, which can travel from the mother’s gut to the fetal brain and cause autism-like behavior by interfering with the neurotransmitter function [40]. Human epidemiological studies have suggested that maternal inflammation could be a reason for ASD. This was confirmed by studies of a mouse model, where pregnant and nonpregnant mice were inoculated with segmented filamentous bacteria or human commensal bacteria that can induce Th17 immune cells or cause maternal inflammation. Pregnant mice secreted interleukin (IL)-1, IL-23, and IL-6, which are stimuli for the induction of Th17 cells to produce IL-17; however, this was not seen in nonpregnant mice [41]. Therefore, the maternal gut microbiome could induce inflammatory cytokines that interfere with the neurodevelopmental process and can lead to neurodevelopmental disorders. Gut dysbiosis, infection during pregnancy, or any health issues in early childhood during development can activate deleterious signal transduction pathways. Pathogen-associated molecular proteins (PAMPs), highly specific specialized proteins present in bacterial lipopolysaccharides, lipoteichoic acid, and peptidoglycan layers of pathogens recognized by the pattern recognition receptor (PRR), are present on gut epithelial cells. Moreover, host immune cells produce inflammatory reactions. PAMP association with PRR results in the activation of downstream signal transduction pathways, such as nuclear factor-κB (NF-κB), mitogen-activated protein kinase (MAPK), and activator protein-1 (AP-1). NF-κB activation results in the production of downstream inflammatory mediators, such as tumor necrosis factor-alpha (TNF-α) and ILs (IL-1, IL-6, and IL-13), and can stimulate the AP-1 transduction pathway. Activation of the AP-1 signal transduction pathway results in the transduction of growth factors, inflammatory proteins, and stress factors. Activation of the MAPK signal transduction pathway results in the activation and release of various inflammatory cytokines, notably IL-17 [42,43].

**Figure 4:**
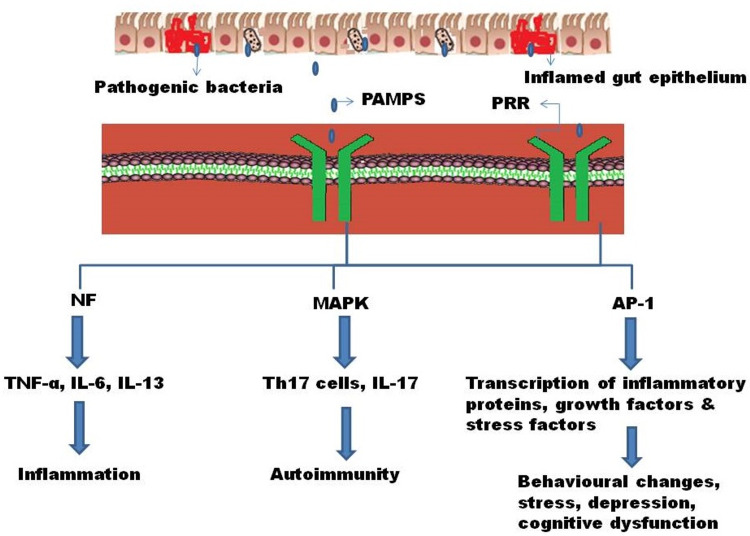
Effect of gut dysbiosis on ASD. PAMPS: pathogen-associated molecular proteins; PRR: pattern recognition receptor; NF: necrosis factor; MAPK: mitogen-activated protein kinase; AP-1: activator protein-1; TNF: tumor necrosis factor; IL: interleukin; Th17 cell: helper T cell 17; ASD: autism spectrum disorder

De Angelis et al. demonstrated that anti-inflammatory *Faecalibacterium* decreased in ASD, whereas increased Enterobacteriaceae and Sutterellaceae were noted [39]. In another study by Hsiao et al.,* B. fragilis* on oral administration improved intestinal integrity, modulated metabolites from the intestine, and improved the symptoms of ASD. Hence, this led to a hypothesis that the gut microbiota can ameliorate the symptoms of ASD. Hence, Hsiao et al. provided oral supplementation of *B. fragilis* to a mouse model and demonstrated improved gut permeability and reduced behavioral changes, as seen in ASD [44]. Certain species, such as *Lactobacillus* and *Bifidobacterium*, and ingestion of probiotics have shown beneficial effects, including improved gut permeability, reduced GI symptoms, and improved cognition and behavior [45]. Figure 4 depicts the effect of gut dysbiosis in ASD.

Headache and Migraine

The human gut microbiome is not only associated with neurodegenerative diseases, such as Parkinson’s disease and Alzheimer’s disease, but also associated with neuropsychiatric illnesses, such as depression and autism disorders. Recently, poor gut health has been linked with migraine attacks [46]. The human gut microbiome is constantly connected to the brain either indirectly via secretion of various neurotransmitters (such as serotonin, dopamine, and noradrenaline), various proinflammatory cytokines (such as IL-1, IL-6, and IL-23), and fatty acids or directly via neural brain connection through the vagus nerve. Any disturbance in gut ecology can affect brain homeostasis and mental health [47].

The direct pathway is rare and can be treated surgically, whereas it is difficult to find a causal relationship to the indirect pathway via neurotransmitters, hormones, food fermentation products/metabolites, inflammatory cytokines, etc.; hence, it is difficult to treat. However, all these factors tend to converge at the gut microbiome [48]. Gut dysbiosis provides an inflammatory environment, leads to an imbalance in neurotransmitter release, and causes metabolites to be released into circulation. For decades, certain foods, such as chocolate and wine, have been known to predispose to migraine attacks, but the gut microbiome composition that ferments these foods is unknown. Recently, several studies have shown their microbial composition and relationship with migraine attacks. In a double-blind, randomized controlled trial by Martami et al., in 40 chronic migraine patients, 14 different probiotic strains were given to one group and a placebo was given to another group. They reported a significant reduction in migraine frequency and severity and concluded that probiotic supplementation could effectively prevent migraine attacks [49]. In a large study, Gonazalez et al. analyzed fecal samples from 1,996 participants with or without migraines. They found that individuals suffering from migraines tend to have more nitrate-producing bacteria than those who do not suffer from migraine attacks [50,51].

Proinflammatory cytokines released by the dysbiosis gut cause inflammation and disrupt the integrity of the gut epithelium. Increased glutamate excitatory neurotransmitters released from the dysbiosis gut may enter the circulation and stimulate nociceptive pain receptors in the CNS and trigeminovascular system that predisposes to migraine attacks [52,53]. This has been demonstrated in several studies. Patients with migraines tend to have higher glutamate and GABA levels. Studies have shown that the dysbiosis gut with predominant *E. coli* and *Enterococcus faecalis* releases neuropeptide Y, substance P, and calcitonin gene-related peptide (CGRP), which can stimulate and induce nociceptive receptors predisposed to migraine attacks [54]. CGRP administration in a mouse model inhibited gastric acid secretion and could precipitate migraine attacks. Probiotic administration significantly reduced migraine attacks by reducing inflammatory cytokines and neurotransmitter modulation [55]. In a recent study, a significant decrease in migraine attacks was seen with probiotic usage compared to placebo. Studies also found deficient/insufficient vitamin D levels in people with migraine compared to control, showing that vitamin D supplementation in the dysbiosis gut significantly reduces harmful inflammatory bacteria, such as *Helicobacter *spp. [56].

Gut dysbiosis in diseases of the bowel

Dysbiosis is common in IBD and is characterized by reduced bacterial diversity with a reduction in Bacteroides and an increase in *E. coli*. *Faecalibacterium prausnitzii* and *Roseburia hominis* are reduced in patients with IBD [57-59]. Alteration of the intestinal microflora is associated with abnormal gut immune responses in genetically susceptible individuals. The predisposing genes of IBD are also related to the recognition of pathogenic microbes [60]. The role of probiotics, antibiotics, and microbial implantation in suppressing inflammation in IBD, especially ulcerative colitis, also supports the role of microbiota in IBD [61].

Inflammatory Bowel Disease

IBD results in inflammatory reactions in the brain, activation of behavioral control areas and the hypothalamic-pituitary-adrenal (HPA) axis, and alteration of the BBB. Proinflammatory cytokines of IBD directly affect the brain via BBB or the vagus nerve, altering neuronal plasticity, microglial activation, and dysregulation of the HPA axis, leading to structural and functional changes in the brain [62]. The dysregulated HPA axis can lead to excess cortisol levels, causing depression in IBD patients [63]. Several animal studies demonstrated increased inflammatory cytokine levels in the hippocampal, cerebral, and hypothalamic regions. There is a correlation between CNS imbalance and psychological behaviors in IBD. It also affects the volume of gray matter and the size of the brain. Agostini et al. reported that patients with Crohn’s disease had decreased gray matter volume in the frontal cortex and anterior cingulated gyrus [64].

Stress has deteriorating effects on IBD by mast cell activation, decreasing the anti-inflammatory pathway and increasing the sympathetic tone. Stress also increases intestinal permeability, leading to superinfection and altered neuronal activity in stress-sensitive areas of the brain [65]. Stress also increases inflammation of the gut by reducing the synthesis and metabolism of SCFAs. Behavioral disorders alter cell signaling, which causes oxidative stress and hypoxia, modifying the microbiological environment of the gut [66]. Psychological symptoms, such as depression and anxiety, are not only common in patients with IBD but they also influence the course of the disease [67].

Irritable Bowel Syndrome

IBS is a common functional disorder characterized by recurrent abdominal pain relieved by defecation. It is common in females, with a prevalence ranging from 10% to 15% [68]. Rome-IV criteria are used to diagnose IBS [69]. IBS is classified into four types as IBS-D (diarrhea-predominant), IBS-C (constipation predominant), IBS-M (mixed pattern), and IBS-U (unclassified). IBS is also associated with other functional disorders such as dyspepsia, chronic pelvic pain, and chronic fatigue syndrome. Psychiatric illnesses, such as anxiety and depression, are highly linked with IBS. Although the exact pathogenesis of IBS is poorly understood, genetic predisposition, food intolerance, visceral hypersensitivity, altered GBA, and dysbiosis may play a role [70].

Normally, an intact epithelial barrier enables the microbes to colonize the intestine and perform symbiosis. This is altered by inflammation, infection, and immune dysregulation, leading to the alteration of the gut microbiome predisposing to IBS by altering the gut immunity and modulating the GBA and gut neuromuscular junction [71]. *Lactobacillus*, *Bifidobacterium*, and Methanobacteriales are decreased and Bacteroides and *E. coli* are increased in IBS [72]. Fungal dysbiosis also indicates visceral hypersensitivity of IBS. Chronic low-grade inflammation along with impaired bowel motility also play a role in IBS. This may be due to infection, dysbiosis, and stress. An increased number of immune cells, such as mast cells and lymphocytes, and increased cytokine production are seen in intestinal biopsies of IBS patients [73]. Proinflammatory cytokines, such as IL-6, TNF-α, and IL-1β, are increased in IBS patients. Additionally, these cytokines are associated with depression and anxiety, suggesting the role of GBA [74]. There is also an association between IL-17 and TNF-α with disease symptoms and quality of life among the different IBS types. Patients with IBS often have altered bowel motility influenced by stress via the GBA. This is due to alterations in serotonin metabolism [75]. Diet plays a major role in pathogenesis by altering gut motility, dysbiosis, GBA, and neuroendocrine actions. Visceral hypersensitivity is commonly caused by gut dysbiosis, alteration in the GBA, diet, psychological factors, and genetic predisposition. Hence, targeting GBA and local neural pathways will be beneficial in the management of visceral hypersensitivity. Anxiety and depression are common comorbidities in IBS patients, suggesting that the brain drives these conditions in patients with IBS [76]. Further, stress aggravates the symptoms of IBS, suggesting the role of brain-derived gut pathogenesis. Nevertheless, GI symptoms appear first in IBS patients, followed by anxiety and depression, suggesting the role of GBA [77]. Management of IBS by probiotics, prebiotics, symbiotics, and antibiotics also suggests the major role of dysbiosis in the pathogenesis of IBS [78]. Fecal microbial transplantation (FMT) plays a major role in the treatment of IBS. Several studies have reported favorable outcomes with FMT in IBS. However, few studies were not in favor of FMT [79-81]. Further studies are needed to assess the role of FMT in the management of IBS.

## Conclusions

The gut microbiome is an integral part of the human health system, comprising dynamic microbes ranging from beneficial microbiota to opportunistic pathogens, and plays an important role in maintaining the health of human beings. Each individual has a unique microbiota that is very diverse and complex in nature. The microbiome starts colonizing the gut immediately after birth and starts connecting to the brain during the developmental process. Any disequilibrium between the gut microbiome and its harmonical relations with normal physiology leads to gut dysbiosis. Factors such as diet, physical inactivity, and drugs affect the gut microbiome.

Gut dysbiosis results in altered neurotransmitter release and affects the GBA, leading to behavioral changes, mood swings, sleep deprivation, depression, and increased anxiety, as seen in Alzheimer’s disease. Moreover, it leads to the deposition of synuclein proteins, leading to neurodegeneration. The study of the changes in the gut microbiome can help in the early diagnosis of Parkinson’s disease and Alzheimer’s disease, and the prevention of gut dysbiosis plays an important role in the treatment of these neurodegenerative diseases. Current evidence suggests that gut dysbiosis is associated with autism, headache, migraine, IBD, and IBS. Physicians need to focus on gut dysbiosis while treating gut and brain disorders and insist upon a diet that helps maintain the gut microbiome. Lastly, the successful treatment of many neurodegenerative, neuropsychiatric, and gut disorders lies in and around the equilibrium of the gut microbiome.
